# Gingival Soft Tissue Integrative Zirconia Abutments with High Fracture Toughness and Low-Temperature Degradation Resistance

**DOI:** 10.34133/bmr.0137

**Published:** 2025-01-23

**Authors:** Qiulan Li, Mianfeng Yao, Yunxu Yang, Bixiao Lin, Hongio Chen, Huixia Luo, Chao Zhang, Yanhao Huang, Yutao Jian, Ke Zhao, Xiaodong Wang

**Affiliations:** ^1^ Hospital of Stomatology, Sun Yat-Sen University, Guangzhou 510055, China.; ^2^ Guangdong Provincial Key Laboratory of Stomatology, Guangzhou 510055, China.; ^3^ School and Hospital of Stomatology, Guangzhou Medical University, Guangzhou 510182, China.; ^4^ Shenzhen Hospital of Integrated Traditional Chinese and Western Medicine, Shenzhen 518104, China.; ^5^ School of Materials Science and Engineering, State Key Laboratory of Optoelectronic Materials and Technologies, Guangdong Provincial Key Laboratory of Magnetoelectric Physics and Devices, Key Lab of Polymer Composite & Functional Materials, Sun Yat-Sen University, Guangzhou 510275, China.; ^6^ Institute of Stomatology, Sun Yat-Sen University, Guangzhou 510055, China.

## Abstract

Low fracture toughness, low-temperature degradation (LTD) susceptibility, and inadequate soft tissue integration greatly limit the application of zirconia ceramic abutment. Integrating the “surface” of hard all-ceramic materials into the gingival soft tissue and simultaneously promoting the “inner” LTD resistance and fracture toughness is challenging. Composite ceramics are effective in improving the comprehensive properties of materials. In this study, we aim to develop a zirconia composite abutment with high “inner” structure stability and “surface” bioactivities simultaneously and to explore the mechanism of performance improvement. Therefore, elongated SrAl_12_O_19_ and equiaxed Al_2_O_3_ were introduced into the zirconia matrix by using the Pechini method. Reinforcements of different shapes can promote the density, reduce the grain size, and increase the phase stability of composite ceramics, which improves the fracture toughness and LTD susceptibility. In addition, the released strontium ions (Sr^2+^), without sacrificing the mechanical properties of the material, could activate the biological capacity of the zirconia surface by activating the M2 polarization of macrophages through the Sr^2+^/calcium-sensing receptor/SH3 domain-binding protein 5 axis, thereby promoting the collagen matrix synthesis of fibroblasts and the angiogenesis of vascular endothelial cells. This successful case proposes a novel strategy for the development of advanced high-strength and bioactive all-ceramic materials by introducing reinforcements containing biofunctional elements into the ceramic matrix. The approach paves the way for the widespread application of such all-ceramic materials in soft-tissue-related areas.

## Introduction

The widespread use of dental implants has raised concerns about the prevalence of peri-implantitis: approximately 19.83% for subjects and 9.25% for implant sites [1]. In addition to osseointegration, robust and stable soft tissue integration is essential for maintaining peri-implant tissue health [2]. The abutment is a critical transmucosal part of the implant that determines the level of soft tissue attachment around the implant. For decades, titanium (Ti) abutments have been regarded as the “gold standard” but are limited by potential cytotoxicity issues and gingival discoloration [3]. In contrast, zirconia abutments are “metal-free” and have excellent biocompatibility, outstanding esthetic properties, and favorable mechanical properties [4]. However, due to its low-temperature degradation (LTD) sensitivity [5] and inadequate soft tissue integration [6], clinicians are hesitant to select zirconia as an abutment material.

LTD in zirconia gained attention in the 2000s when a large number of zirconia femoral heads were recalled due to fractures shortly after implantation. LTD has been recently found in dental zirconia restorations [7], albeit not known widely. It is characterized by spontaneous t–m phase transformation in the presence of water accompanied by 3% to 5% volume expansion, leading to an extensive grain lift and pullout [8], which in turn results in increased roughness, microcrack generation, and decreased mechanical properties, which ultimately shorten the lifespan of zirconia materials [5]. Although efforts have been made to improve the LTD sensitivity of zirconia, several issues are persistent. For example, increased yttria content to high translucent partially stabilized zirconia (PSZ) or altered stabilizer to cerium oxide (CeO_2_) (Ce-TZP) can effectively reduce the t–m phase transition and improve the LTD resistance; however, both approaches compromise the strength of zirconia [9,10]. Nonetheless, there is an avenue for zirconia ceramics with improved LTD resistance.

Furthermore, the soft tissue around the zirconia abutment contains a lower amount of gingival fibroblasts (3% to 5% vs. 15%) and a relatively low density of vasculature (only the supra-periosteal blood vessel system) compared to periodontal gingiva around the natural tooth, which is similar to Ti abutment [11]. Fewer fibroblasts and vasculature weaken the attachment of the connective tissue layer to the abutment surfaces, resulting in apical proliferation of epithelial cells and compromised soft tissue integration [12]. Subsequent to implant placement, various cells accumulate at the surgical site and promote healing; their “race to invade” at the abutment–mucosa interface after implantation can determine the fate of the dental implant [12]. Among these, macrophages have aroused important attention since they can rapidly recruit to the injury site after implantation, and their unique plasticity responds to environmental stimuli to improve tissue healing and regeneration [12]. Macrophages are divided into 2 phenotypes: M1 (pro-inflammatory) polarization and M2 (anti-inflammatory) polarization. M2 macrophages play a critical role in soft tissue healing by secreting a series of growth factors to regulate fibroblasts and vascular endothelial cells and promote their adhesion, migration, and proliferation [13]. Recent studies have indicated that biomaterial-mediated optimization of macrophage phenotypes represents an attractive strategy for enhanced soft tissue integration [14].

Several studies have focused on improving the bioactivity of zirconia abutments through surface coating and surface modification, taking into account wettability, morphology, and roughness [2]. However, cutting processes and subtractive treatment cause defects and microcracks on the zirconia surface, increasing the risk of mechanical complications [15]. Since modifying the inert zirconia surface is difficult, adhesive failure of the functionalized coating may be inevitable [16]. Importantly, the surface modification of the zirconia abutment might not improve the LTD sensitivity of its internal structure.

In order to improve the comprehensive properties of zirconia, developing zirconia composites by adding different reinforcements is trending [17]. Circular Al_2_O_3_ is the most commonly used reinforcement in zirconia, which could limit matrix grain growth, thus improving LTD resistance [18]. The biological activity of the material can be improved with the addition of bioactive elements; among these, strontium (Sr), one of the vital trace elements, might be preferred [19]. Some studies have shown that Sr promotes skin wound healing by inducing M2-type polarization of macrophages [20]. On the other hand, Sr forms an elongated type of hexa-aluminate SrAl_12_O_19_, which prevents the propagation of cracks by crack deflection and bridging mechanisms, thereby increasing fracture toughness [21]. Although previous studies have investigated whether the addition of Al_2_O_3_ and SrAl_12_O_19_ to Ce-TZP improves fracture toughness and LTD resistance [22], their role in 3 mol.% yttria-stabilized tetragonal zirconia (3Y-TZP) has not yet been elucidated. Since abutment materials require higher strength and less translucency (unlike a crown), 3Y-TZP is more suitable as an abutment material than 5Y-PSZ [9]. This will open avenues to exploring its possibility as zirconia abutment.

In the present study, we aim to develop a zirconia composite abutment with high “inner” structure stability and “surface” bioactivities simultaneously and to explore the mechanism of performance improvement. Therefore, we added both equiaxed Al_2_O_3_ and elongated SrAl_12_O_19_ into 3Y-TZP to improve LTD resistance by using the restriction effect of differently shaped reinforcements on matrix grains while promoting M2-type polarization of macrophages by releasing the bioactive ions Sr^2+^. This strategy could also improve its fracture toughness, thus developing a new zirconia abutment with high mechanical properties, LTD resistance, and sufficient soft tissue sealing. Firstly, zirconia composite ceramics with different reinforcement concentrations were synthesized using the Pechini method, and their effects on LTD resistance and fracture toughness were studied to determine the optimal concentrations of the reinforcements. Then, we analyzed their connective tissue integration efficiency as abutment materials by dissecting the induction effect of macrophages and its potential activation pathways. In the present study, we developed zirconia composites from the perspective of dental implant abutment, which broadens its application in the dental field and paves the way for extensive applications in other medical fields that require biological activity and mechanical properties.

## Materials and Methods

### Zirconia composite synthesis

A modified Pechini one-pot method was used to synthesize the zirconia composite [23]. Zr(NO_3_)_4_·5H_2_O (>99.99% purity), Y(NO_3_)_3_·6H_2_O (>99.5% purity), Al(NO_3_)_3_·9H_2_O (>99% purity), and Sr(NO_3_)_2_ (>99% purity) were used as starting powders (Sigma-Aldrich, St. Louis, MO, USA). All powders were solubilized in distilled water for equivalent proportions of SrAl_12_O_19_ and Al_2_O_3_ in sintered materials, which were 0 to 15 vol.% (inspired by Palmero et al. [22]), and the yttria content was 3 mol.%. Citric acid monohydrate and ethylene glycol were added to the solution as chelating agents. Consequently, a wet gel was obtained at ≤110 °C by constant stirring and evaporation of moisture from the suspensions on the magnetic stirrer. The wet gel was dried at 140 °C for 24 h. The composition was heated in a muffle furnace at 600 °C to break down its nitric compounds. The resulting powder composition was dispersed in ethanol and ball milled for 24 h at 300 r/s in a planetary ball mill machine (QXQM-4, Tianchuang Powder Technology Co., Ltd, Changsha, China) with zirconia spheres (2.0 mm in diameter). Subsequently, the powder composition was separated from ethanol by a rotary evaporator, dried at 60 °C for 24 h, and compacted by axial pressing at a pressure of 100 MPa and cold isostatic pressing at 250 MPa to fabricate a green body with a 10-mm diameter and a 1.5-mm thickness. The green body was sintered in air at 1,500 °C for 3 h at a heating rate of 5 °C/min to obtain the final composite sample. Finally, we obtained 6 groups of samples: ZrO_2_, ZrO_2_ + 3% Al_2_O_3_ + 3% SrAl_12_O_19_, ZrO_2_ + 5% Al_2_O_3_ + 5% SrAl_12_O_19_, ZrO_2_ + 8% Al_2_O_3_ + 8% SrAl_12_O_19_, ZrO_2_ + 10% Al_2_O_3_ + 10% SrAl_12_O_19_, and ZrO_2_ + 15% Al_2_O_3_ + 15% SrAl_12_O_19_. In addition, ZrO_2_ + 8% Al_2_O_3_ and ZrO_2_ + 8% SrAl_12_O_19_ were synthesized as controls. The commercially available dental zirconia (3Y-TZP, ST, Upcera, Shenyang, China) of the same size obtained by diamond saw cutting was used as the control group. Samples were polished to 3 μm using diamond lapping film (D-512X, Grish, Beijing, China).

### Characterization

The density of specimens was measured according to the Archimedes method with deionized water as the immersion medium [22]. The phase analysis of sintered samples was carried out by x-ray diffractometry (XRD; D8 ADVANCE diffractometer, Bruker, Billerica, MA, USA) using nickel-filtered Cu Kα radiation and energy in the 2*θ* range of 20° to 80°, with a scanning rate of 2 °/min and a step size of 0.02° according to ISO 13356:2008. The microstructure of the samples was observed under field emission scanning electron microscopy (SEM; Nova Nano-SEM 450, FEI, Hillsboro, OR, USA) after thermal etching at 1,350 °C for 1 h according to ISO 13356:2008. The grain size was measured using the ImageJ software. The disks for transmission electron microscopy were prepared using standard techniques, and thin foils were produced using the ion milling technique (focused ion beam, Helios 450S dual beam, FEI). These specimens were examined under a high-resolution transmission electron microscope (HR-TEM; Titan Themis 200, FEI), and an energy-dispersive x-ray spectrometer (EDS; super-X, Bruker) was used to detect the elemental composition of various crystal phases [22].

The surface roughness of the zirconia composite before and after immersion in the cell medium was examined by laser confocal microscopy (LSM 700, Zeiss Jena, Oberkochen, Germany). In addition, the specimen was immersed in Dulbecco’s modified Eagle medium (DMEM) (1 ml/cm^2^) for 1, 7, 14, and 28 d, and the Sr^2+^ concentration was measured by inductively coupled plasma–mass spectrometry (Agilent 8900 ICP-MS, Agilent, Beijing, China).

### Mechanical properties

Fracture toughness was determined according to the Vickers indentation method with a 10-s loading time and a 10-kgf constant load (DuraScan-20, Struers, Denmark) [24]. The hardness and elastic modulus were measured by nanoindentation on a nanoindentation instrument (Nano Indenter G200, Keysight Technologies, Santa Rosa, CA, USA) using a continuous stiffness measurement module; the maximum indentation depth was 1,000 nm. The flexural strength by the ball-on-3-ball test was measured on a universal testing machine (Instron E3000, Instron, Boston, MA, USA) at a loading rate of 0.5 mm/min [25].

### LTD analysis

LTD was conducted at 134 °C and 0.2 MPa for 8, 16, 32, and 64 h. Subsequently, the increase in the monoclinic phase was determined by XRD and calculated using the Garvie–Nicholson method according to ISO 13356:2008. The surface roughness was measured by atomic force microscopy (XE-100, Park Systems Corporation, South Korea) before and after LTD. The depth of LTD at the cross-section of specimens was measured by SEM.

### Cell culture

The murine-derived macrophage cell line RAW 264.7, L929 cells, human umbilical vein endothelial cells (HUVECs), and primary human gingival fibroblasts (HGFs) were kind gifts from the Xiangya Hospital Center for Molecular Medicine (Changsha, China). RAW 264.7 cells were cultured on the surface of the zirconia composite or in conditioned medium (CM) in which the zirconia composite was immersed for 7 d or in SrCl_2_ solution with a concentration gradient of Sr^2+^ (0, 5, 50, and 500 μg/l), respectively. In addition, RAW 264.7 cells were treated with 20 mmol of the calcium-sensing receptor (CaSR) antagonist NPS-2143 (SB-262470A, MedChemExpress, NJ, USA) for 24 h and cultured in zirconia composite immersion. Subsequently, the cells were cultured in DMEM supplemented with 10% fetal bovine serum and 1% penicillin/streptomycin (HyClone, Thermo Fisher Scientific Inc., Waltham, MA, USA) at 37 °C in a humidified atmosphere under 5% CO_2_ [26].

### Cell viability and proliferation test

To estimate the in vitro cellular biocompatibility, L929 cells were cultured in zirconia composite immersion and 3Y-TZP. Cell Counting Kit-8 (Dojindo, Kyushu Island, Japan) was used to assess the cell viability and proliferation of L929 cells. The optical density of the cells cultured for 1, 2, 3, 4, and 5 d was measured at 450 nm on a microplate reader (BioTek, VT, USA) [26].

### Cell immunocytochemistry

The secretion and deposition of inducible nitric oxide synthase (iNOS) and CD206 by RAW 264.7 cultured in zirconia composite immersion were visualized by immunofluorescence staining [26]. All samples were fixed in 4% paraformaldehyde for 15 min, permeabilized with 0.1% Triton X-100 for 5 min, and subsequently blocked with 5% bovine serum albumin in phosphate-buffered saline (PBS) for 30 min. After blocking, the samples were incubated overnight with rabbit anti-iNOS antibody (ab15323, Abcam, Cambridge, UK) and rabbit anti-CD206 antibody (ab64693, Abcam) at 4 °C, followed by incubation with the secondary antibody (1:1,000 Alexa Fluor 488 goat anti-rabbit IgG [H + L], Bioss, Beijing, China) in the dark for 1 h. Finally, the cells were counterstained with 4′,6-diamidino-2-phenylindole for 10 min, and images were captured using a confocal laser scanning microscope (TCS SP8, Leica, Vizsla, Germany).

### Quantitative reverse transcription polymerase chain reaction

Total RNA was extracted using TRIzol (Thermo Fisher Scientific) according to the manufacturer’s instructions. A PrimeScript RT Reagent Kit (RR074A, TaKaRa, Kyoto, Japan) was used to reverse-transcribe the RNA into complementary DNA. Quantitative reverse transcription polymerase chain reaction was performed on a C1000 Touch thermal cycler (Bio-Rad, Hercules, CA, USA) using SYBR Green Mix (Bio-Rad). All samples were assayed in triplicate with 3 independent experiments. The primer sequences are listed in Supplementary Materials (Table S1).

### Cell chemotaxis assay

RAW 264.7 cells were treated in zirconia composite immersion with or without NPS treatment for 48 h, rinsed with PBS, and cultured in fresh medium for 24 h. Then, the CM was collected. An equivalent of 100 μl of CM was added to the lower chamber as a chemoattractant, and HGFs or HUVECs (1 × 10^5^) were added to the upper chamber. Subsequently, cells were incubated in this system at 37 °C for 24 to 48 h. After removing the noninvading cells with cotton swabs, migrated cells were fixed with pre-cooled methanol and stained with 2% Giemsa solution. Finally, stained cells were counted in at least 3 randomly selected fields at ×100 magnification under a microscope (Zeiss) to minimize the bias.

### Co-culture of RAW 264.7 and HGF cells

Transwell inserts (0.4 μm; Corning, USA) were used for co-culture. RAW 264.7 cells in zirconia composite immersion with or without NPS treatment for 48 h were rinsed with PBS, cultured in fresh medium for 24 h, and introduced into the upper chamber. HGF cells (1 × 10^5^) were added to the lower chamber and co-cultured with the treated RAW 264.7 cells (5 × 10^4^). After 24 h, total RNA was isolated from HGF cells and analyzed by quantitative reverse transcription polymerase chain reaction to detect the expression of *COL1A1* and *COL3A1*.

### Tubule formation assay for HUVECs

A 24-well plate was coated with Matrigel (BD Biosciences, Bedford, MA, USA) at 37 °C for 30 min. HUVECs (1 × 10^5^) were incubated in 100 μl of CM for 12 h before imaging. The capillary tubes were quantified under a ×100 bright-field microscope (Zeiss) by measuring the total length of the completed tubule structure. Three independent experiments were required for each treatment.

### RNA sequencing and bioinformatics analysis

Total RNA was extracted from RAW 264.7 cells cultured in Al_2_O_3_/ZrO_2_ and Al_2_O_3_/SrAl_12_O_19_/ZrO_2_ composite immersions, respectively. The sequencing library was sequenced on a NovaSeq 6000 platform (Illumina, San Diego, CA, USA) by Shanghai Personal Biotechnology Co., Ltd. Raw data were generated in FASTQ format and further transformed into read counts and fragments per kilobase million reads data for downstream analysis. All statistical analyses were conducted using R Studio 4.0 (The R Foundation, USA). DESeq2 was used to identify the differentially expressed genes (DEGs), defined as fold change ≥2 and *P* value ≤0.05. Two public microarray datasets (GSE53321 and GSE72518) were retrieved from the Gene Expression Omnibus database to identify the M2-macrophage-polarization-related genes. The intersections between DEGs and M2-macrophage polarization-related genes were analyzed by using the Venn diagram.

### Plasmid and siRNA transfection

RAW 264.7 cells were transfected with small interfering RNAs (siRNAs) targeting SH3 domain-binding protein 5 (SH3BP5) or negative control siRNA (Guangzhou Ribo Life Science Co., Ltd, Guangzhou, China), according to the manufacturer’s protocols. *SH3BP5* was overexpressed in pcDNA3.1–SH3BP5 (Qingke, Beijing, China) for 48 h, and plasmid pcDNA3.1Xflag-v5-ccdB (Qingke) was used as the negative control before detection with ViaFect (E4981, Promega, Madison, WI, USA) [26].

### Western blotting

Total protein lysates were obtained using a lysis buffer. The supernatant of the lysate was collected by centrifugation to determine the protein concentration using a bicinchoninic acid assay. Proteins were separated by sodium dodecyl sulfate–polyacrylamide gel electrophoresis and transferred to polyvinylidene fluoride membranes. Then, the membranes were probed with anti-iNOS antibody (ab15323, Abcam), rabbit anti-CD206 antibody (ab64693, Abcam), and α-tubulin (sc-23948, Santa Cruz, Dallas, TX, USA) overnight at 4 °C. The immunoreactive bands were visualized using horseradish peroxidase (HRP)-conjugated secondary antibodies [26].

### Oral implantation and topical application of HRP

Screw-type pure Ti implants with cemented ceramic abutments were prepared for the in vivo experiments. Ethical approval for this study was granted by the Ethics Committee of the Sun Yat-Sen University (approval number A29-227-0). The upper first molar on the left side of Sprague–Dawley male rats (5 weeks old, each weighing 150 to 200 g) was extracted under anesthesia, followed by implantation after 4 weeks. The HRP test was used to evaluate the strength of soft tissue sealing at 2 and 4 weeks of healing [11]. The oral mucosa surrounding the implant was collected after the maxilla with the implant was immersed in 4% paraformaldehyde and demineralized in 5% tetrasodium ethylenediaminetetraacetate. All specimens were immersed in 20% sucrose at 4 °C overnight, embedded in an optical cutting temperature compound (Sakura, Tokyo, Japan), and sliced into 10-μm buccal–palatal sections with a cryostat (Thermo Fisher Scientific) at −20 °C.

### Histological analysis and immunohistochemistry

The sections were stained with hematoxylin–eosin and Masson’s trichrome according to the standard procedure for histological observation. Briefly, the sections were blocked with 3% hydrogen peroxide and bovine serum albumin and incubated at 4 °C with anti-iNOS antibody (ab15323, Abcam), rabbit anti-CD206 antibody (ab64693, Abcam), and rabbit anti-vascular endothelial cadherin antibody (ab205336, Abcam), respectively, overnight; alkaline phosphatase-conjugated goat anti-rabbit IgG (SA00002-2, Proteintech, Beijing, China) was used as the secondary antibody. Specific proteins were stained using the BCIP/NBT Alkaline Phosphatase Color Development Kit (Sigma-Aldrich), according to the manufacturer’s guidelines. On the other hand, HRP histochemistry was performed using 3,3′-diaminobenzidine staining [27].

### Statistical analysis

Statistical analysis was performed using the Student *t* test between 2 groups and one-way analysis of variance and post hoc Tukey’s test in multiple comparisons using the SPSS 20.0 software (IBM SPSS). *P* < 0.05 was considered statistically significant. All data are expressed as mean ± standard deviation (SD).

## Results and Discussion

### Composite microstructural features

**Fig. 1.**
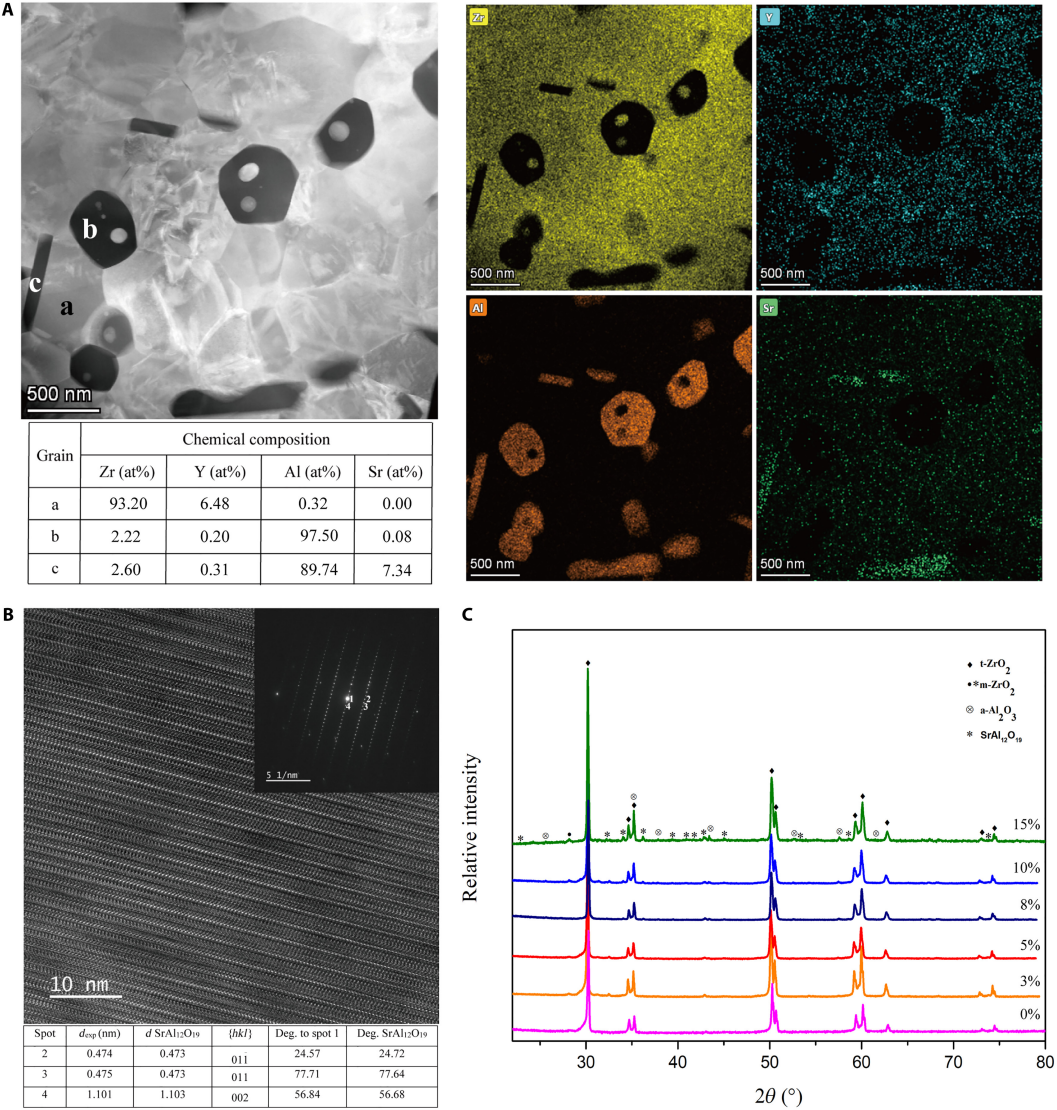
Characterization of zirconia composite. (A) Transmission electron microscope (TEM) image and the corresponding energy-dispersive x-ray spectrometer (EDS) maps of the sintered zirconia composite with 8 vol.% SrAl_12_O_19_ and 8 vol.% Al_2_O_3_; (B) high-resolution TEM (HR-TEM) micrograph of a SrAl_12_O_19_ grain (fast Fourier transformation [FFT] is shown in the inset in the figure) and the corresponding FFT analysis (below); (C) x-ray diffractometry (XRD) patterns of sintered zirconia composite with varied contents of SrAl_12_O_19_ and Al_2_O_3_.

HR-TEM analysis provided an in-depth insight into the microstructure (bright regular shape, dark equiaxial shape, and elongated grains) of a sintered sample of composite zirconia with 8 vol.% SrAl_12_O_19_ and 8 vol.% Al_2_O_3_ (Fig. 1A). Also, the chemical composition of the particles with different morphologies and phase contrast was characterized by an EDS nanoprobe focused on the grains (Fig. 1A, a, b, and c). The table enclosed in Fig. 1A shows that the corresponding atomic compositions were in agreement with their nominal compositions; however, a low yttrium and zirconium amount was observed in b and c grains, which could be attributed to a matrix effect [22]. The dark equiaxial grain at point b was corroborated as alumina. Moreover, the atomic Sr:Al ratio of 7:90 (=1:12.8) at point c (dark elongated grain) confirmed that the aluminate phase composition was similar to that of SrAl_12_O_19_ within the instrumental error (the thin foil geometry might affect uncontrolled absorption). The brighter grain (point a) was pure zirconia stabilized with yttria. The atomic ratio of yttria was 6.48%, indicating that the zirconia stabilization degree was slightly higher than the expected value (3 mol.% Y_2_O_3_), but still within the instrumental error. Figure 1A also shows the EDS maps of the zirconia composite distinctly, confirming the presence of zirconium and yttrium inside the brighter grains, strontium only in the elongated grains, and aluminum in both dark elongated and rounded grains.

The fast Fourier transformation (FFT) from HR-TEM micrographs was exploited for phase identification and compared with the lattice distance using Powder Diffraction File (PDF) cards. Figure 1B is a representative of the HR-TEM image of a strontium aluminate grain and the associated FFT (inset in the figure). The indexation corresponds to the hexagonal SrAl_12_O_19_ phase according to the angles between lattice planes and lattice distances of the PDF card (00-026-0976), respectively. FFT on a zirconia and an alumina grain confirmed the crystallography of the tetragonal-ZrO_2_ (01-070-4426) phase (Fig. S1) and Al_2_O_3_ (PDF no. 04-004-5434) (Fig. S2).

The XRD patterns of the sintered materials for composite zirconia with 0, 3, 5, 8, 10, and 15% concentrations of SrAl_12_O_19_ and Al_2_O_3_, respectively, are shown in Fig. 1C. The images show the presence of tetragonal zirconia phases (PDF no. 704431) and the α-Al_2_O_3_ (PDF no. 9770) and SrAl_12_O_19_ phases (PDF no. 69020). With increasing concentration of reinforcements, the small diffraction peaks at 2*θ* = 28.2° representing the m phase decrease gradually, and there is almost no m phase at 8 vol.%. However, it is also up-regulated in a reinforcement-concentration-dependent manner at >8% concentration.

These results suggest that the zirconia composite with the desired composition was produced successfully using the Pechini method. Also, the yttrium amount inside the zirconia grains was tailored by the addition of Al_2_O_3_ and SrAl_12_O_19_ [28]. The higher the yttrium content in the zirconia lattice, the greater the stabilizing effect of yttrium in the zirconia lattice and the lower the monoclinic fraction.

**Fig. 2.**
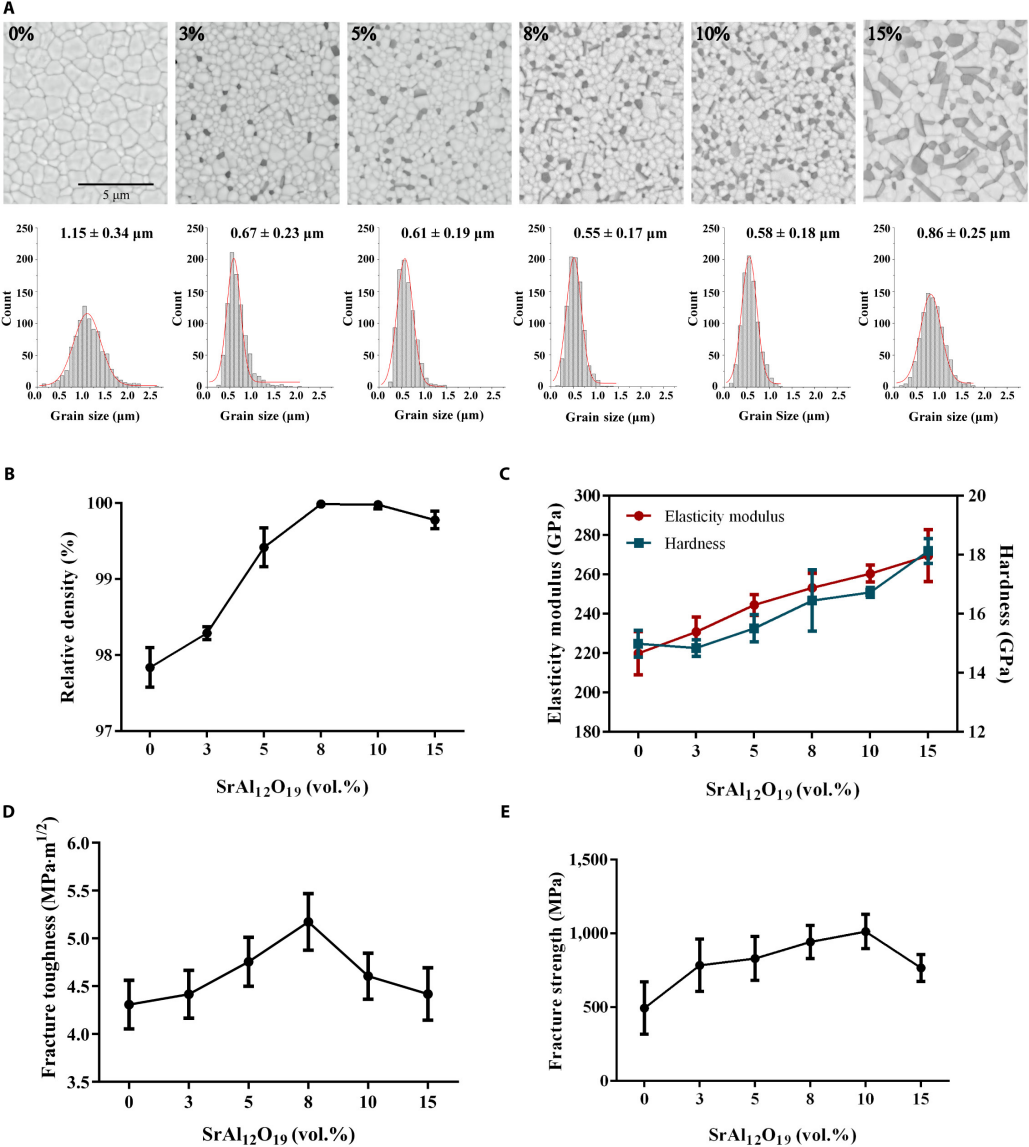
Microstructure and mechanical properties of zirconia composite with different contents of SrAl_12_O_19_ and Al_2_O_3_. (A) Scanning electron microscopy (SEM) images and grain size distribution of ZrO_2_; (B) relative density; (C) elasticity modulus and hardness; (D) fracture toughness; (E) fracture strength.

Representative SEM micrographs and grain size distributions of zirconia composite materials with different reinforcement concentrations are shown in Fig. 2A. All phases inside the composite materials are homogeneous at SrAl_12_O_19_ and Al_2_O_3_ concentrations below 10 vol.%. However, at an increased concentration of reinforcements of 15%, the grains of reinforcements can overlap and clump in the zirconia composite. In addition, the grain size of zirconia was correlated with the reinforcement content; the grain size decreased with increasing reinforcement content from 0 to 10 vol.%. The lowest grain size of 0.55 ± 0.17 μm was obtained in zirconia with 8 vol.% reinforcements. Nonetheless, the addition of 15 vol.% reinforcements increased the grain size of zirconia again. The trend of Al_2_O_3_ grain size is the same as that of zirconia (Fig. S3), while the aspect ratio of SrAl_12_O_19_ increased first, followed by a decrease with increasing concentration of the reinforcement (Fig. S4).

The relative density of zirconia without reinforcements was 97.84% (Fig. 2B). The density of zirconia composites increased gradually with the increasing content of the reinforcements, reaching the maximum density at 8 and 10 vol.% (>99.9%). However, the density of the composites decreased as the reinforcement content increased further to 15 vol.%.

These results suggest that the appropriate amount of SrAl_12_O_19_ and Al_2_O_3_ is favorable for the formation of dense composite zirconia. The network structure formed between platelike SrAl_12_O_19_ and the low sintering ability of SrAl_12_O_19_ platelets might be attributed to this phenomenon resulting from the inhibition of interlayer diffusion of atoms [29]. A low SrAl_12_O_19_ content effectuates a high sintering resistance of SrAl_12_O_19_ platelets and a very low sintering rate compared to that of ZrO_2_ grains. Thus, the SrAl_12_O_19_ platelet acts as a spacer or friction to prohibit the migration of the grain boundaries and facilitate densification [30]. However, when the addition exceeds the critical value, the SrAl_12_O_19_ elongated grains and Al_2_O_3_ equiaxial grains may overlap mutually or stack, which impairs the densification kinetics by introducing a large number of pores in the host matrix; this phenomenon retards the acquisition of dense ceramics [31]. The asymmetric shape of the SrAl_12_O_19_ platelets also creates interstitial spaces between the particles, forming closed pores [21]. This change in material density shows a close correlation with grain size data. The grain size of ZrO_2_ and Al_2_O_3_ decreases gradually, and the aspect ratio of SrAl_12_O_19_ increases gradually when the material density increases in a reinforcement-concentration-dependent manner; however, when the density decreases with a further increase in the concentration of the reinforcements, their grain size displays an opposite trend. These results were attributed to the fact that the presence of the reinforcements distributed along the boundaries can produce a pinning effect and inhibit the grain growth of the matrix. Nonetheless, the decreased density with the increasing concentration of reinforcements reduces the restriction between the crystals, thus limiting the pinning impact and promoting the abnormal growth of the ZrO_2_ grains. These results are consistent with those presented by Tang et al. [30].

### Mechanical properties

Figure 2C shows that the hardness and elastic modulus values increased continuously with increasing reinforcements, achieving the maximum values of 18.13 ± 0.42 and 269.55 ± 13.22 GPa at a content of 15 vol.%, respectively. Among the 3 kinds of grains, Al_2_O_3_ is the hardest phase in the composite [31]. Therefore, increasing the Al_2_O_3_ content increases the overall hardness of the composites. In addition, Al_2_O_3_ shows the highest elastic modulus among the phases. Hence, according to the rule of mixtures, the elastic modulus value of the composites rose with the increasing Al_2_O_3_ content, and the maximum value of elastic modulus was attained for the 15 vol.% composite sample with the highest content of the Al_2_O_3_ phase.

Fracture toughness values obtained from Vickers indentation testing of the zirconia composites (Fig. 2D) indicate that the fracture toughness was maximum at 8 vol.% (5.17 ± 0.30 MPa·m^1/2^) and then decreased with increasing concentrations of reinforcements. Based on the indentation, it can be found that the increasing concentration of the reinforcement first shortens the crack length of the rhombic corner, which elongates subsequently (Fig. S5). Although the indentation fracture method is disputable when used in phase-transforming materials, our previous findings [10] suggest that the method is feasible when used for comparative purposes. Our previous study showed that the fracture toughness of zirconia with varied yttria contents in the dental market was within 2.42 to 3.53 MPa·m^1/2^ [10]. Therefore, the fracture toughness of the zirconia composite developed in this study improved by about 46% with favorable fracture strength. According to the requirements of ISO 6872:2015 (dentistry—ceramic materials), the fracture toughness of dental ceramics greater than 5 MPa·m^1/2^ is classified as grade 5, which can cover almost all kinds of dental usage scenarios.

Figure 2E shows the results of fracture strength obtained from the ball-on-3-ball test. Incorporation of SrAl_12_O_19_ and Al_2_O_3_ into the zirconia matrix increased the fracture strength, reaching a maximum at 10 vol.%, followed by a gradual decrease. The fracture strengths of zirconia with 0, 3, 5, 8, 10, and 15 vol.% were 494.89 ± 176.62, 784.58 ± 177.16, 830.64 ± 148.28, 942.77 ± 112.21, 1,012.96 ± 116.58, and 766.61 ± 90.43 MPa, respectively.

Some studies have established that the predominant mechanisms underlying the enhanced mechanical properties of platelet-reinforced ceramic composites are crack bridging, deflection, and platelet pullout [30,32]. In addition, the high aspect ratio of SrAl_12_O_19_ platelets and the weak interphase bonding strength between the reinforcements and matrix grains are conducive to widespread crack deflection, resulting in increased fracture energy dissipation and crack propagation path. However, the increased densification resistance of the composite with high reinforcement additions produces a large number of pores in the ZrO_2_ matrix. These pores might act as stress concentrators and counteract the reinforcing effect, decreasing fracture toughness and flexural strength. As a result, the increasing reinforcement content initially augments the mechanical properties of the ceramic composites, which then decreases slightly when the reinforcement additions exceed the critical value. This variation and peak mechanical properties are in good agreement with previous findings on platelet-reinforced ceramic composites [30].

However, the increased mechanical properties come at the expense of translucency. In order to improve the translucency of zirconia as a crown material, the content of the stabilizer Y_2_O_3_ is often increased at the cost of mechanical properties [33]. However, zirconia composite ceramics are opaque due to the presence of multiple grain interfaces. Fortunately, because of the coverage of the gingiva and crowns, the abutment can meet esthetic requirements as long as it is not as dark as the metal material.

### LTD analysis

**Fig. 3.**
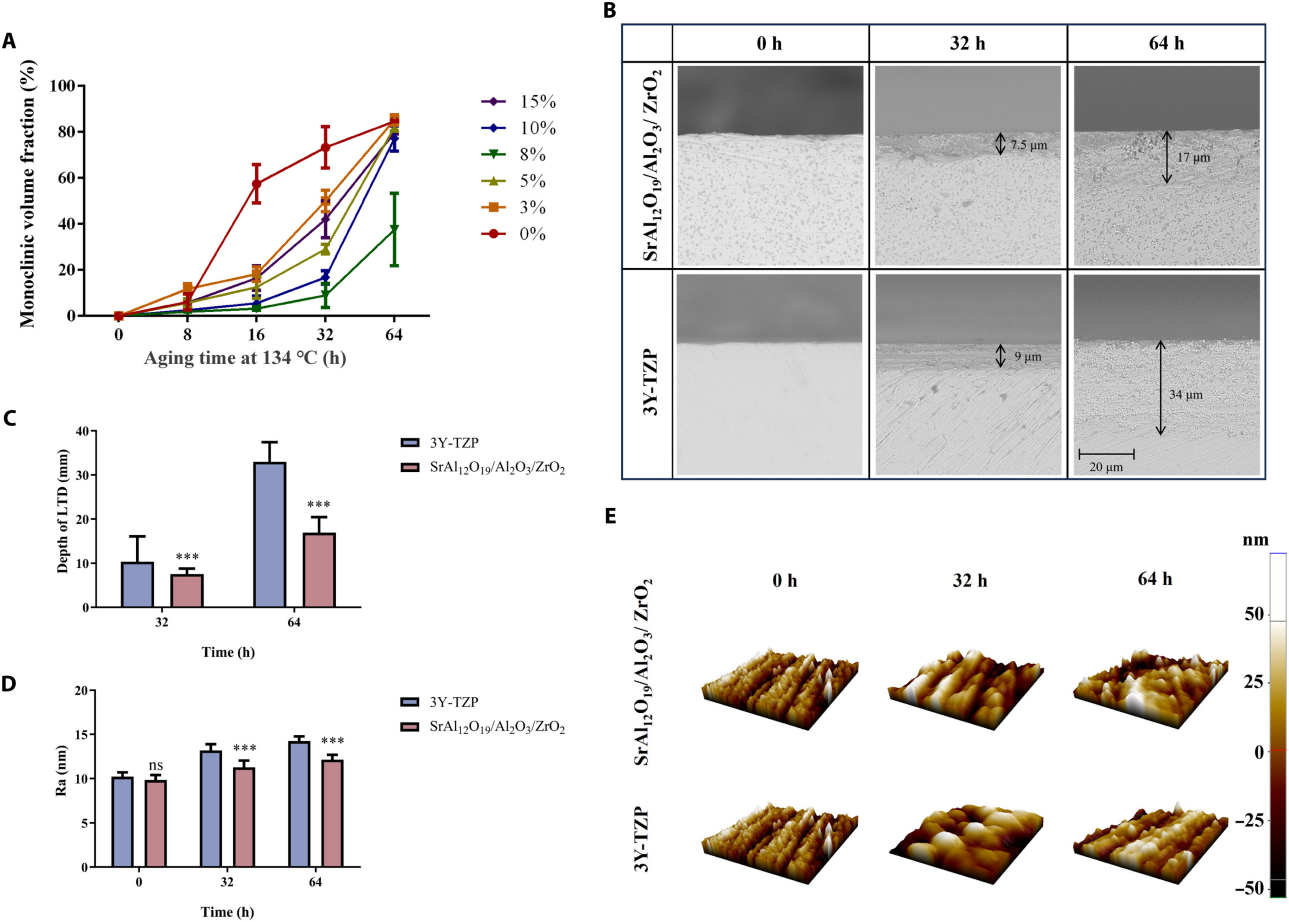
Low-temperature degradation (LTD) analysis of zirconia composite. (A) The monoclinic volume fractions of zirconia composites with different contents of reinforcements; (B and C) the depth of phase transformation region at cross-sections; (D and E) surface roughness of the commercial 3 mol.% yttria-stabilized zirconia (3Y-TZP) and zirconia composite with 8 vol.% SrAl_12_O_19_ and 8 vol.% Al_2_O_3_. The Student *t* test was used between 3Y-TZP and the zirconia composite. ***P < 0.001; ns, no significant difference.

The zirconia composites were treated at 134 °C and 0.2 MPa for the LTD test at 8, 16, 32, and 64 h. The results of the monoclinic phase transformed from the tetragonal phase during LTD are presented in Fig. 3A. The fraction of m phase after 16 h of zirconia without reinforcements was significantly higher than that of zirconia composite reinforced with SrAl_12_O_19_ and Al_2_O_3_. The increase in the reinforcements from 0 to 8 vol.% flattened the aging kinetic curve steadily, indicating a decrease in the rate of LTD; however, with a further increase in the reinforcement content, the LTD rate accelerated gradually. After the 64-h treatment, the m-phase volume fraction of all samples was approximately 80% except for that of the sample of the 8 vol.% group, which was <40%.

Next, we compared the depth of the phase transformation region at the cross-sections of commercial 3Y-TZP and zirconia composites containing 8 vol.% SrAl_12_O_19_ and 8 vol.% Al_2_O_3_. Figure 3B and C show that the addition of SrAl_12_O_19_ and Al_2_O_3_ significantly reduces the vertical transformation propagation from the surface of the composite. After 32-h exposure, the zirconia composite had about 7.5-μm transformed layers, while the commercial 3Y-TZP showed signs of transformation at a depth of 10 μm. Strikingly, after 64 h of exposure, the transformed layer depth was maximal at 33 μm on the 3Y-TZP material and only 17 μm on the composite sample. The surface roughness obtained from atomic force microscopy of the zirconia composite and 3Y-TZP exposure for 32 and 64 h is presented in Fig. 3D and E. The results indicate that the roughness of zirconia composite is 11.3 and 12.1 nm, which is significantly lower than that of 3Y-TZP, i.e., 13.2 and 14.2 nm after 32- and 64-h exposure, respectively.

These findings implied that SrAl_12_O_19_ and Al_2_O_3_ reinforcements inhibited the propagation rate of t–m transformation in the zirconia matrix and increased the resistance of zirconia to LTD. This phenomenon can be explained by the following reasons: Firstly, the addition of reinforcements decreases the grain size of zirconia, and the larger grain size results in higher local tensile stress, where t-zirconia is more likely to be further converted to m-zirconia, thus reducing the LTD resistance [34]. Another possible mechanism for the improved hydrothermal stability of the zirconia composite might be the increased cohesion strength between the grain boundaries [35]. LTD starts from the surface and leads to microcracks at the grain boundaries due to phase-transformation-induced volume expansion [36]. Thus, a strong cohesion between ZrO_2_ grains’ boundaries in the presence of SrAl_12_O_19_ and Al_2_O_3_ hindered the formation of microcracks and impeded the propagation of aging-induced t–m phase transformation [36]. In addition, alumina and aluminates lead to a change in elastic strain energy related to t–m transformation and an increase in matrix stiffness, which consequently hinders t–m transformation [37].

Based on the effects of various reinforcement contents on the microstructure, mechanical properties, and LTD resistance of zirconia composite ceramics mentioned above, we speculated that zirconia with 8 vol.% SrAl_12_O_19_ and 8 vol.% Al_2_O_3_ has comprehensive properties. This newly developed zirconia composite has approximately 46% higher fracture toughness and approximately 40% higher LTD resistance compared to commercially available dental zirconia materials [10]. Therefore, this composite ceramic was used in subsequent experiments.

### Structural stability of zirconia composite ceramics in a body fluid environment

**Fig. 4.**
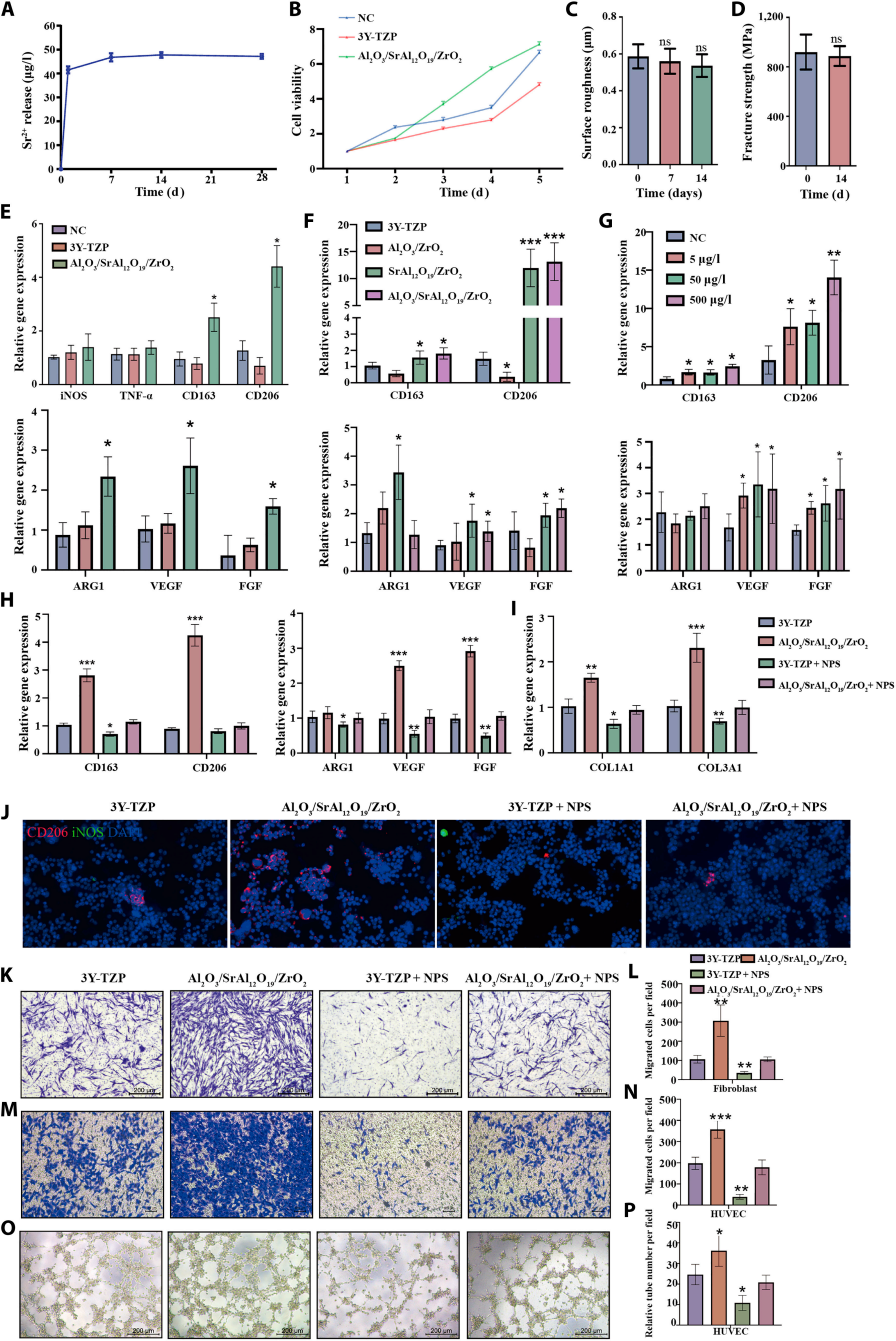
Effect of zirconia composite ceramics on macrophage polarization. (A) Concentrations of Sr^2+^ released from zirconia composites immersed in Dulbecco’s modified Eagle medium (DMEM). (B) Cell proliferation of L929 cells. (C) Surface roughness of zirconia composites after immersion for different durations. (D) Fracture strength of zirconia composites after immersion for 14 d. The relative expression of genes related to macrophage polarization, angiogenesis, and fiber formation (basic fibroblast growth factor [bFGF]) in macrophages cultured on the surface of a zirconia disk (E), in different zirconia immersions (F), in SrCl_2_ solution with a gradient concentration of Sr^2+^ (G), and in zirconia immersions with or without NPS pretreatment (H). (I) Relative expression of collagen-synthesis-related genes of fibroblasts co-cultured with macrophage-conditioned culture medium. (J) Immunofluorescence staining of macrophage polarization markers of macrophages cultured in different zirconia immersions. Representative images (K) and quantitative results (L) of migrated fibroblasts co-cultured with macrophage-conditioned culture medium. Representative images (M) and quantitative results (N) of migrated vascular endothelial cells co-cultured with macrophage-conditioned culture medium. Representative images (O) and quantitative results (P) of the tube formation of vascular endothelial cells co-cultured with macrophage-conditioned culture medium. Data are shown as mean ± SD (*n* = 3 biological replicates); post hoc Tukey’s test was used for multiple comparisons. *P < 0.05; **P < 0.01; ***P < 0.001; ns, no significant difference. NC, negative control; iNOS, inducible nitric oxide synthase; TNF-α, tumor necrosis factor-alpha; ARG1, arginase-1; VEGF, vascular endothelial growth factor; FGF, fibroblast growth factor; COL1A1, collagen, type I, alpha1; COL3A1, collagen, type III, alpha1; DAPI, 4′,6-diamidino-2-phenylindole; HUVEC, human umbilical vein endothelial cell.

We hypothesized that the zirconia composite ceramic may promote connective tissue integration around the abutment by releasing Sr^2+^. Therefore, we measured the levels of Sr^2+^ release of the zirconia composite with 8 vol.% SrAl_12_O_19_ and 8 vol.% Al_2_O_3_ in DMEM. Figure 4A shows that the concentration of Sr^2+^ in the medium increased rapidly on day 1 of immersion, reaching saturation at 47 μg/l on day 7. Furthermore, after immersion for 28 d, the concentration of Sr^2+^ in zirconia with 3.%, 5, and 10 vol.% reinforcements were 66.10, 64.28, and 42.77 μg/l, respectively. These concentrations are orders of magnitude lower than the effective concentration of Sr^2+^ reported previously [38]. Reportedly, a concentration of Sr^2+^ >0.21 mg/ml in drinking water caused hepatotoxicity in embryonic zebra fish [39]. In another study, calcium phosphate cement containing 10 μg/ml Sr^2+^ was highly toxic to mouse gingival fibroblast cells [40]; this concentration was much higher than that measured in the zirconia composite immersion. Therefore, the release of Sr^2+^ from the composite material is considered safe. The results of the cell proliferation assay confirm that the cell compatibility of the zirconia composite did not differ significantly from that of the control group on day 5 (Fig. 4B). Moreover, the release of this small amount of Sr^2+^ does not affect the structural stability of the material, because the surface roughness (Fig. 4C), mechanical properties (Fig. 4D), surface topography, and contact angles (Fig. S6) of the material are not altered after immersion. These results indicate that zirconia composite ceramics can safely release a very small amount of Sr^2+^ in the body fluid environment without affecting the structural stability of the material.

### Effect of zirconia composite ceramics on macrophage polarization

In order to explore the effect of composite ceramics on macrophages, we cultured macrophages on the surface of composite and commercial 3Y-TZP zirconia ceramics. We measured the expression of polarization- as well as angioblastic- and fibroblast-related genes. Interestingly, the expressions of M2-polarization-related genes (*CD163* and *CD206*), angioblastic genes (*ARG1* and *VEGF*), and the fibroblast-related gene (*FGF*) were significantly increased in the composite ceramic group, while no significant difference was detected in the expression of M1-type polarization-related genes *iNOS* and tumor necrosis factor-alpha (*TNF-α*) (Fig. 4E).

To further investigate whether the composite ceramics on macrophages effectuate through Sr, macrophages were cultured in the ceramic immersion of the 4 groups: commercial 3Y-TZP, zirconia composite with 8 vol.% Al_2_O_3_, zirconia composite with 8 vol.% SrAl_12_O_19_, and zirconia composite with 8 vol.% SrAl_12_O_19_ and 8 vol.% Al_2_O_3_. We observed that only the group with SrAl_12_O_19_ showed high expression of *CD163*, *CD206*, *ARG1*, *VEGF*, and *FGF* (Fig. 4F). In addition, the macrophages were cultured in a gradient of SrCl_2_ according to the Sr^2+^ concentration measured in the composite ceramic immersion. The results show that only 5 μg/l of Sr^2+^ can sufficiently induce M2 polarization and promote the expression of *VEGF* and *FGF* on the macrophages; the gene expression level increased in a Sr^2+^ concentration-dependent manner (Fig. 4G). Thus, it can be deduced that Sr^2+^ in the composite ceramics induces the M2 polarization of macrophages, which was in accordance with previous studies [41].

Sr^2+^ has chemical and biological properties, and its valence states are similar to those of Ca^2+^. Sr^2+^ can also activate CaSR in different bone cells in bone tissue engineering to promote osteoblastogenesis and inhibit bone resorption activity by modulating downstream signaling pathways [42]. Reportedly, Ca^2+^ mediates the M2 polarization of macrophages through bonding with CaSR at the cell surface [43]. Therefore, to investigate whether Sr^2+^-induced M2 polarization was mediated via CaSR, macrophages were pretreated with the CaSR inhibitor NPS and cultured in zirconia composite immersion. The results show that the up-regulated expression of *CD163*, *CD206*, *ARG1*, *VEGF*, and *FGF* genes in macrophages was inhibited after NPS pretreatment (Fig. 4H and J), indicating that Sr^2+^ induces M2 polarization through the CaSR of macrophages. Therefore, zirconia composite immersion with 8 vol.% SrAl_12_O_19_ and 8 vol.% Al_2_O_3_ was used in the subsequent experiments.

M2-polarized macrophages activate the functions of fibroblasts and vascular endothelial cells, the 2 main repair cell types involved in the healing process of soft tissue around the abutment. Our study also showed that M2-polarized macrophages activated by the composite ceramic promote the chemotaxis and the expression of collagen-related genes (*COL1A1* and *COL3A1*) in fibroblasts (Fig. 4I, K, and L). In addition, the co-culture with M2 macrophages increased the chemotaxis and the tubular structure formation of HUVECs (Fig. 4M to P). However, when pretreated with the CaSR inhibitor NPS, the effect of Sr^2+^ on fibroblasts and endothelial cells was inhibited.

Based on these findings, we conclude that zirconia composite ceramics can release extremely small amounts of Sr^2+^ ions without affecting their overall structural stability. These ions induce the M2 polarization of macrophages through CaSR, and the M2 macrophages stimulate the function of fibroblasts and vascular endothelial cells.

### Soft tissue sealing around zirconia composite ceramic abutments

**Fig. 5.**
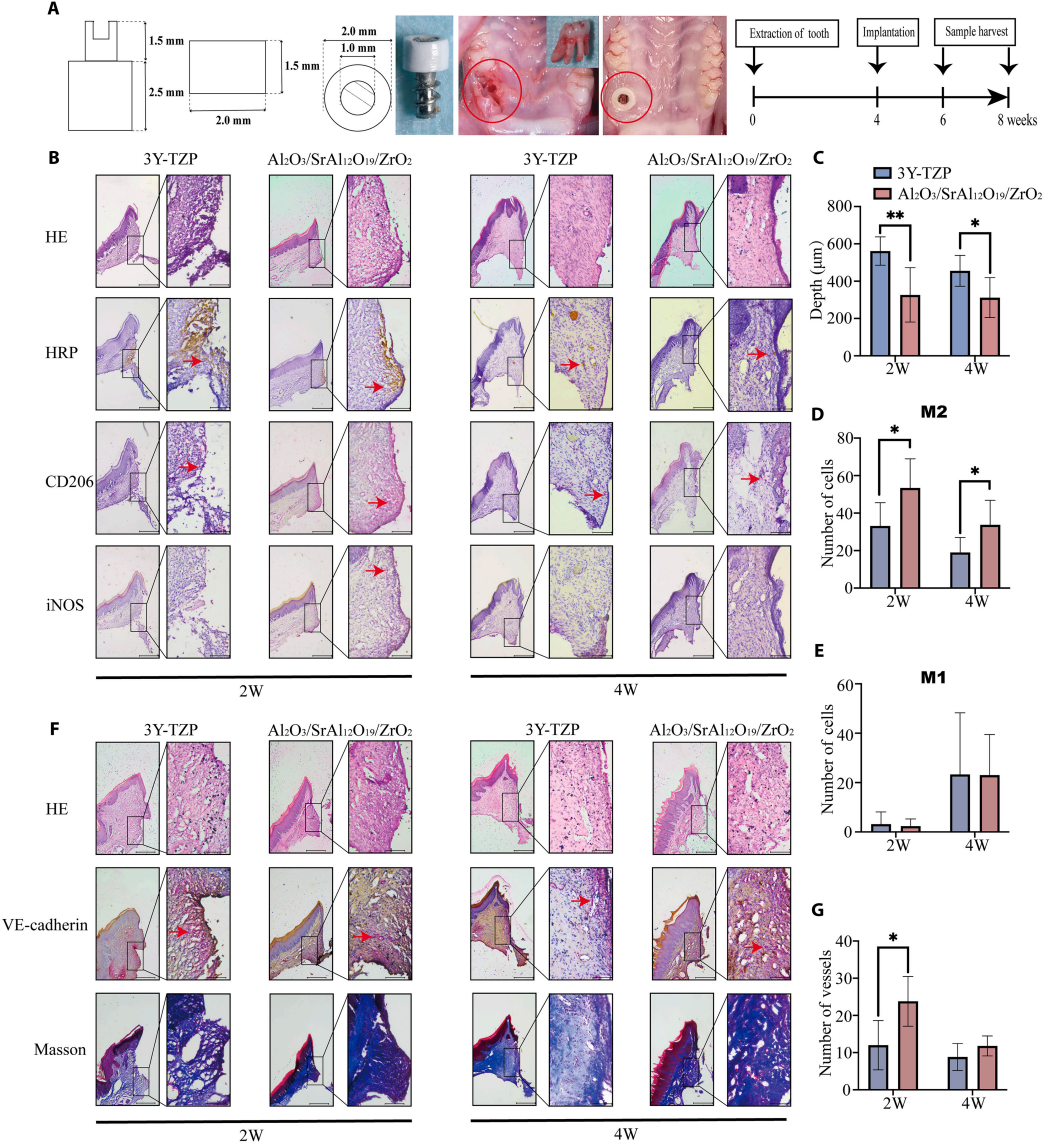
Soft tissue sealing around zirconia composite ceramic abutments. (A) Design of the implant and abutment and the process of implantation. (B) Hematoxylin–eosin (HE) and immunohistochemical staining of horseradish peroxidase (HRP; brown staining, red arrow) and macrophage-polarization-related proteins (red staining, red arrow) in the connective tissue around the abutment after healing for 2 and 4 weeks. (C) Quantitative results of HRP penetration depth. Quantitative results of M2 macrophages (D) and M1 macrophages (E). (F) HE staining, immunohistochemical staining of vascular markers (red staining, red arrow), and Masson staining in the connective tissue around the abutment after healing for 2 and 4 weeks. (G) Quantitative results of vascular density. The bar at left represents 200 μm, and the bar at right represents 50 μm. Data are shown as mean ± SD (*n* = 3 biological replicates); the Student *t* test was used between 3Y-TZP and the zirconia composite. *P < 0.05; **P < 0.01. 2W, 2 weeks; 4W, 4 weeks; VE-cadherin, vascular endothelial cadherin.

Next, we implanted the zirconia composite ceramic abutment and commercial 3Y-TZP abutment into the maxillary bone of rats to investigate the surrounding soft tissue sealing. The HRP test was used to evaluate the performance of soft tissue sealing; the deeper the penetration of HRP from the gingival margin, the worse the sealing. The implantation procedure is illustrated in Fig. 5A. Figure 5B and C shows that the HRP penetration depth around the composite abutment is shallower than that of the commercial 3Y-TZP at 2 and 4 weeks after implantation. Moreover, the number of cells expressing the M2-polarization-related gene *CD206* was significantly higher at 2 and 4 weeks after implantation, while the number of cells expressing the M1-polarization-related gene *iNOS* did not differ markedly (Fig. 5D and E). These results indicate that the M2 polarization of macrophages might be responsible for improved soft tissue sealing around the zirconia composite ceramic abutment.

A lower number of gingival fibroblasts and a relatively low vasculature density are the 2 main reasons for weak soft tissue sealing around the abutment [11]. A previous study reported that M2 macrophages can promote soft tissue healing by activating vascular endothelial cells and fibroblast functions [13]. Herein, we observed angiogenesis and collagen production in the connective tissue around the abutment. The number of vessels in the connective tissue around the composite abutment increased significantly at 2 weeks after implantation, while no significant difference was detected in the control group after 4 weeks. Moreover, at both 2- and 4-week time points post-implantation, the collagen content in the soft tissues in the experimental group was higher than that in the control group (Fig. 5F and G). These findings indicate that the M2 macrophages induced by the composite abutment improve connective tissue sealing by promoting the formation of blood vessels and the production of collagen, which correspond to the results of the in vitro cell assays.

### Mechanism of macrophage M2 polarization induced by Sr^2+^ released from composite zirconia

**Fig. 6.**
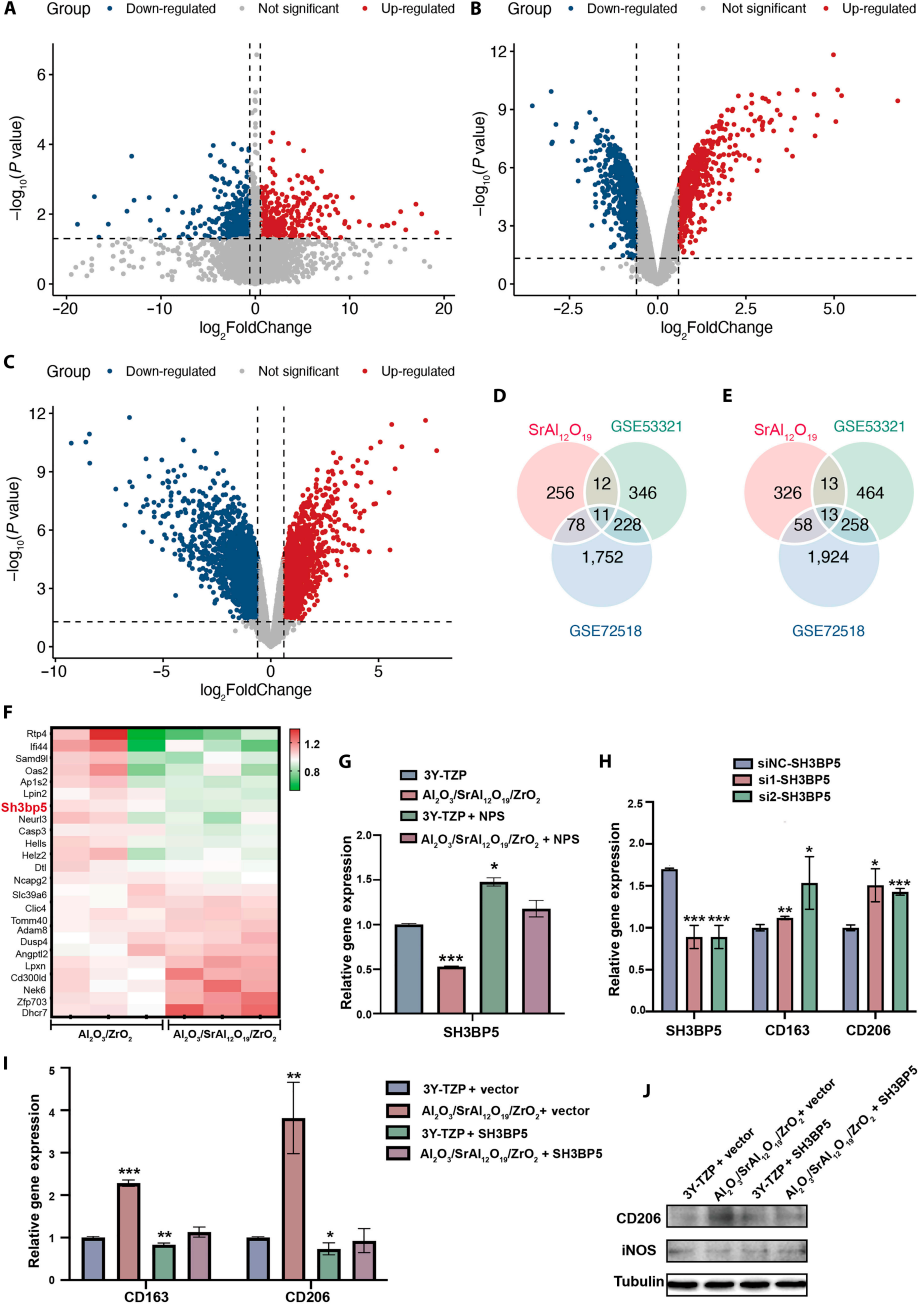
Mechanism of macrophage polarization induced by Sr^2+^ released from composite zirconia. (A) Differentially expressed genes (DEGs) of macrophages treated with zirconia-composite-immersed solutions. DEGs related to M2 macrophage polarization of GSE53321 (B) and GSE72518 (C) datasets. Venn diagram of up- (D) and down-regulated genes (E) in the 3 datasets. (F) Heatmap of DEGs in the 3 datasets. (G) Relative expression of the *SH3BP5* gene in macrophages cultured in zirconia-composite-immersed solutions with or without NPS pretreatment. (H) The relative expression of the *SH3BP5* gene and M2-macrophage-polarization-related genes in macrophages with the *SH3BP5* gene silenced. (I) Relative expression of M2-macrophage-polarization-related genes in *SH3BP5*-gene-overexpressed macrophages cultured in zirconia-immersed solutions. (J) Expression level of macrophage-polarization-related proteins in *SH3BP5*-gene-overexpressed macrophages cultured in zirconia-immersed solutions. Data are shown as mean ± SD (*n* = 3 biological replicates); Tukey’s test was used for multiple comparisons *P < 0.05; **P < 0.01; ***P < 0.001.

In order to further investigate the mechanism underlying Sr^2+^-induced M2 polarization of macrophages, we cultured the macrophages in the zirconia composite immersion with 8 vol.% SrAl_12_O_19_ and 8 vol.% Al_2_O_3_ and zirconia composite immersion with 8 vol.% Al_2_O_3_. RNA sequencing identified 582 DEGs, including 256 up- and 326 down-regulated genes (Fig. 6A). To further identify the genes associated with M2 macrophages, we obtained public datasets GSE53321 and GSE72518 from the Gene Expression Omnibus database; the DEGs are shown in Fig. 6B and C. Then, the intersection between the up- and down-regulated genes in the 3 datasets retrieved 11 up- and 13 down-regulated genes (Fig. 6D and E). A thorough review of the literature on the correlation among these genes, macrophage polarization, and CaSR prompted us to focus on the *SH3BP5* gene among the down-regulated genes (Fig. 6F).

SH3BP5, a protein in the mitochondrial outer membrane, is a binding target and substrate of c-Jun N-terminal kinase (JNK), which impairs mitochondrial function by intramitochondrial signaling pathways and promotes reactive oxygen species production [44]. The mitochondrial dysfunction led to the inhibition of the M2 polarization of macrophages [45]. Therefore, we propose that the down-regulation of the *SH3BP5* gene may be responsible for the Sr^2+^-induced M2 polarization of macrophages. To confirm this hypothesis, we measured the expression of the *SH3BP5* gene and cultured the macrophages in composite ceramic immersion. The results showed a decrease in the expression of *SH3BP5* gene; however, this down-regulation was suppressed by the CaSR inhibitor NPS (Fig. 6G). On the other hand, *SH3BP5* gene silencing in macrophages increased the expression of *CD163* and *CD206* (Fig. 6H), while the expression of these genes decreased when the *SH3BP5* gene was overexpressed in macrophages cultured in composite ceramic immersion (Fig. 6I and J). Collectively, these results indicate that Sr^2+^ inhibits the expression of the *SH3BP5* gene in macrophages through CaSR, thereby promoting the M2 polarization of macrophages.

### Implications of advanced “LTD resistance–high toughness–soft tissue integration” zirconia materials

**Fig. 7.**
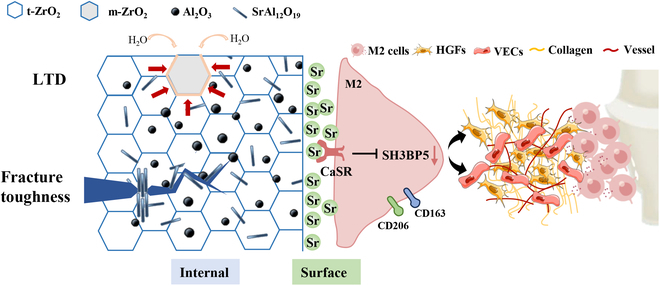
The schematic shows the mechanism in which Al_2_O_3_ and SrAl_12_O_19_ in the zirconia composite abutment modulate the internal microstructure to improve fracture toughness, LTD resistance, and surface soft tissue integration. VECs, vascular endothelial cells.

The low fracture toughness, LTD susceptibility, and inadequate soft tissue integration of zirconia ceramics are the main reasons that prevent the materials from being the first choice of clinicians. Also, integrating the “surface” of hard all-ceramic materials with the gingival soft tissue and “inner” LTD resistance and fracture toughness is challenging. In this study, we applied the strategy of composite ceramics to introduce reinforcement-containing biofunctional elements into the zirconia matrix. This improved the soft tissue integration of the “surface” of the material through the release of Sr^2+^ while regulating the “internal” structure of the material (Fig. 7). Although the ceramic composite principle is successful in this study, the improvement in the internal and external functions of the material is yet to be elucidated, necessitating an in-depth understanding of the underlying mechanism. Based on this knowledge, the researchers can develop advanced all-ceramic materials to precisely modulate the key functional cells around their surface and the key structure network inside, thus overcoming the mechanical and biological shortcomings of the materials.

This preliminary study showed that Sr^2+^ down-regulates the *SH3BP5* gene through CaSR. However, the CaSR-mediated down-regulation of SH3BP5, which, in turn, leads to the M2 polarization of macrophages, needs further exploration. A previous study reported that the activation of SH3BP5 is dependent on the activator protein 1 (AP-1) response element in its promoter region [46]. The AP-1 transcription factor is a dimeric complex composed of JUN, FOS, MAF, and ATF protein family members and is regulated by various cellular pathways, including Ca^2+^-related pathways [47]. In addition, SH3BP5 is related to mitochondrial dysfunction by JNK pathways [44]; this mitochondrial dysfunction suppresses the M2 polarization of macrophages [45]. Therefore, in the future, it can be carried out that after the binding of Sr^2+^ to CaSR, the AP-1 transcription factor is activated to down-regulate the expression of SH3BP5, which in turn leads to the mitochondrial dysfunction through the JNK pathway, and then induce macrophage M2 polarization. In this way, the mechanism by which Sr^2+^ induces macrophage M2 polarization will be fully understood. Also, a thorough study of the mechanism of Sr^2+^-induced macrophage polarization is essential to apply Sr^2+^ in soft-tissue- as well as hard-tissue-related biomaterials.

In addition to its role in soft tissue healing, Sr^2+^ also activates various signaling pathways in bone cells, thereby promoting the function of osteoblasts and inhibiting osteoclast activities [48]. Therefore, the composite ceramics developed in this study might have a critical positive role in bone tissue integration, rendering them suitable for perforation materials that require both osseointegration and soft tissue integration, for example, dental implants. The tissue-level dental implant with soft tissue integration in the upper part and bone tissue integration in the lower part facilitates open healing (one-time healing) and avoids the injury caused by the second surgery. Other biomedical applications would also benefit from the superior hard and soft tissue integration properties of this material, such as perforating implants, artificial limbs, bone-anchored hearing aids, and external fracture fixators, which should be anchored in the bone tissue and extend into the soft tissue. However, the osseointegration ability of the ceramic materials developed in this study must be investigated. Moreover, because of the friction with bone tissue during implantation, the friction and wear properties of this material also need to be further studied.

Furthermore, other bioactive elements, such as lanthanum (La^3+^), can also be applied to similar strategies. La also forms an elongated type of lanthanum hexa-aluminate (LaAl_11_O_18_) that can regulate the microstructure network to improve LTD resistance and fracture toughness similar to those of SrAl_12_O_19_ [18]. In addition, the antihyperphosphatemia, anti-inflammatory, and osteoblast-enhancing effects of La^3+^ [49] improve the bioactivities of the materials. In addition, other bioactive elements, such as calcium or magnesium ions, may also be a promising option. They have excellent bioactivity, biocompatibility, antibacterial and bacteriostatic properties, dimensional stability, and moisture tolerance. However, calcium aluminate (CaAlO_4_) is more commonly used in cement for dental or bone repair due to its low mechanical strength [50]. Although magnesium aluminate (MgAl_2_O_4_) spinel is characterized by high flexure strength, high hardness, and excellent transparency, few studies have added it to zirconia ceramics. Therefore, the selection of bioactive elements needs to consider balance between mechanical and biological properties. Taken together, we present a putative example to improve the overall properties of all-ceramic materials simultaneously. We should bear in mind that introducing biofunctional elements can modulate the surface bioactivity of all-ceramic soft tissue materials, and the reinforcements formed by the biofunctional elements could manipulate the inner microstructure, which, in turn, improves the mechanical properties of the composite.

Due to the important roles of macrophages in soft tissue healing, we have limited research subjects on macrophages. However, this does not mean that macrophages are the only cells involved. The Sr^2+^-wound healing response should be more complex. Other immune cells, such as neutrophils, lymphocytes, and mast cells, should participate in this process and require further investigation. In addition, it should be noted that only the biological performance of zirconia with 8 vol.% SrAl_12_O_19_ and 8 vol.% Al_2_O_3_ was studied, although there was a small difference in the amount of Sr^2+^ released from zirconia with different reinforcement concentrations. This does not mean that the biological performance of other concentrations is not important. The effect of reinforcement concentration on the biological performance of zirconia is also worth exploring. Furthermore, prolonged effects of Sr^2+^ release on both local tissue and implant stability are lacking, although the short-term data indicate that zirconia composite ceramics can safely release a very small amount of Sr^2+^ in the body fluid environment without affecting the structural stability of the material.

## Conclusion

The soft tissue integration of the “surface” of the ceramic abutment and “internal” structure stability were improved by introducing reinforcement-containing biofunctional elements into the zirconia matrix. The applied Al_2_O_3_ and SrAl_12_O_19_ improved the LTD sensitivity and fracture toughness of zirconia by promoting densification, decreasing the grain size, and increasing phase stability. The released Sr^2+^ could down-regulate the expression of SH3BP5 in macrophages through CaSR without sacrificing the mechanical properties of materials, inducing M2 polarization of macrophages, promoting the collagen matrix synthesis of fibroblasts and angiogenesis of vascular endothelial cells, and ultimately leading to stable soft tissue integration of zirconia. Thus, the present study provided a compelling perspective for the development of all-ceramic materials with internal mechanical properties and biointerface–soft tissue integration ability for clinical applications.

## Data Availability

Data will be made available on request.
