# Supplementary material for: Gingival Soft Tissue Integrative Zirconia Abutments with High Fracture Toughness and Low-Temperature Degradation Resistance
Source: Biomater Res. 2025 Jan 23;29:0137. doi: 10.34133/bmr.0137 (PMC11756602; doi:10.34133/bmr.0137)
Supplement: Supplementary 1 — Figs. S1 to S6 Table S1 [file bmr.0137.f1.docx]

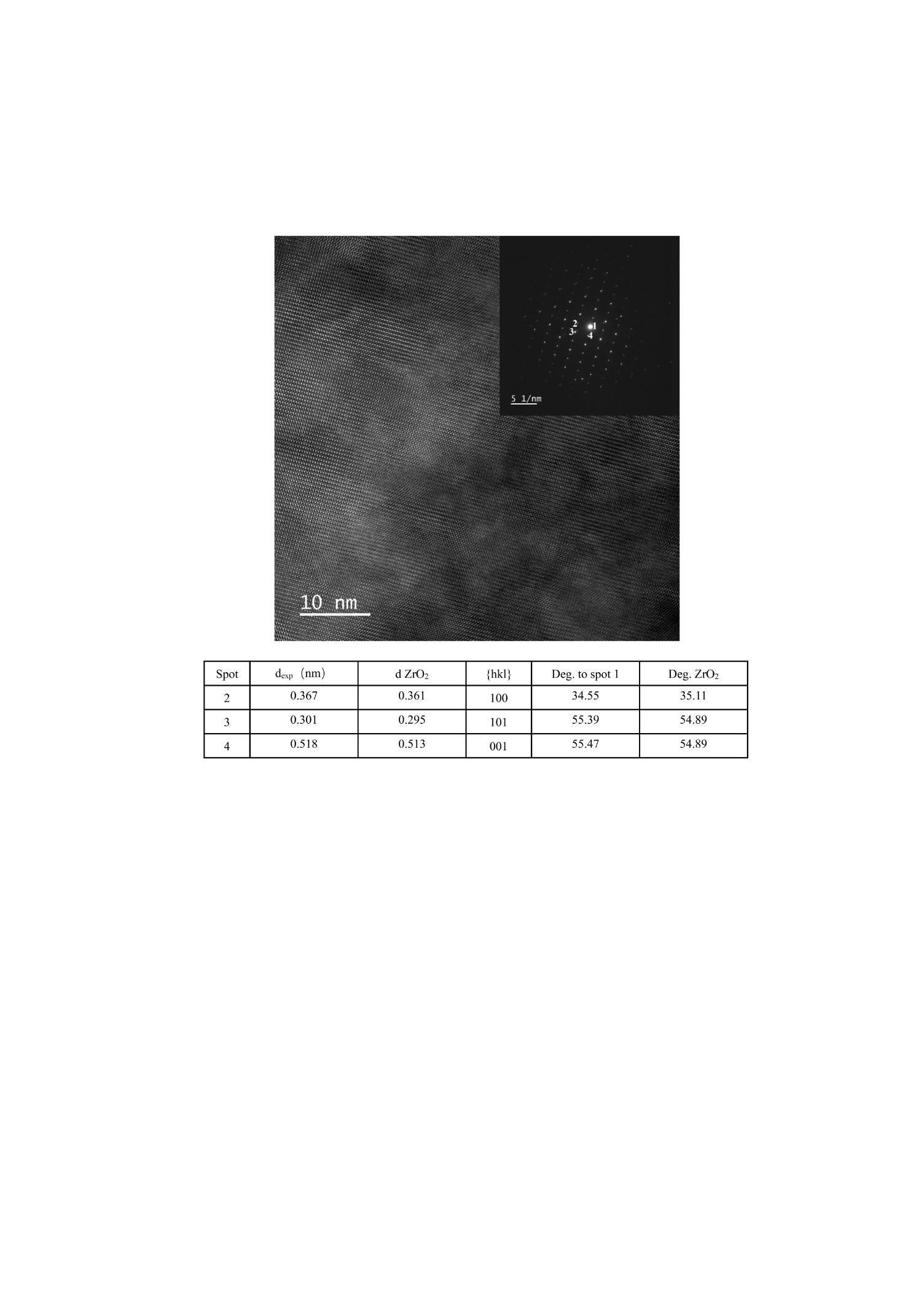


SFig. 1 HRTEM micrograph of a ZrO_2_ grain (FFT was inset to the figure) and the corresponding FFT analysis (below)


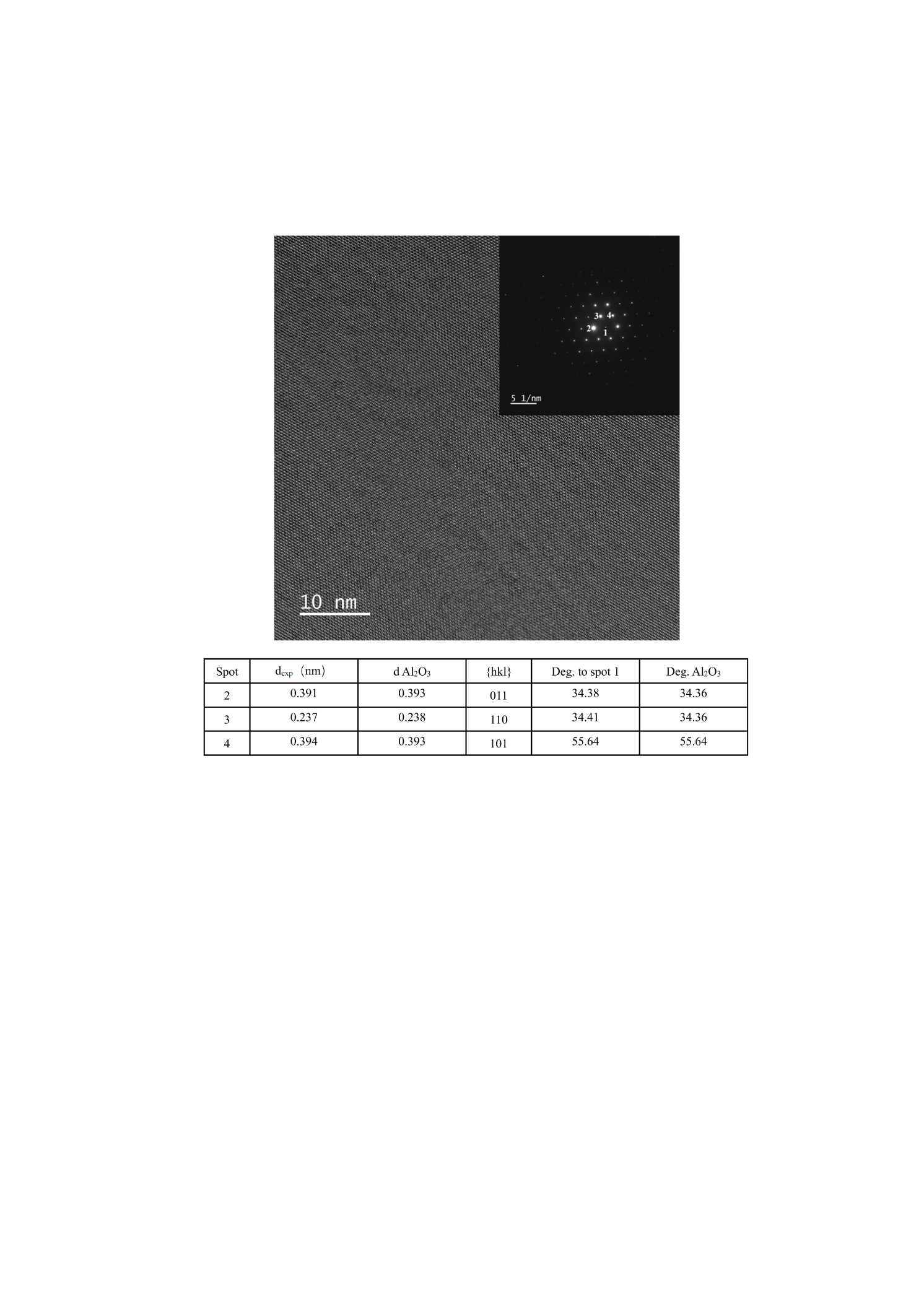


SFig. 2 HRTEM micrograph of a Al_2_O_3_ grain (FFT was inset to the figure) and the corresponding FFT analysis (below).


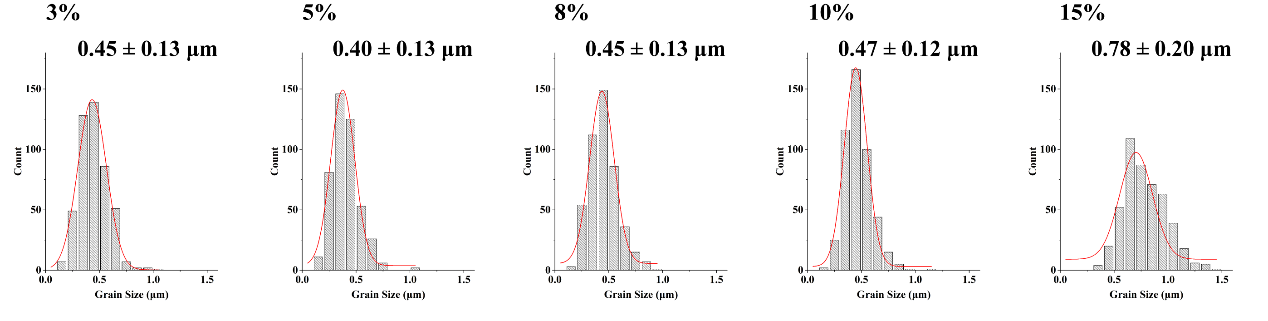


SFig. 3 Grain size distributions of Al_2_O_3_ grain in zirconia composites with different reinforcements concentration.


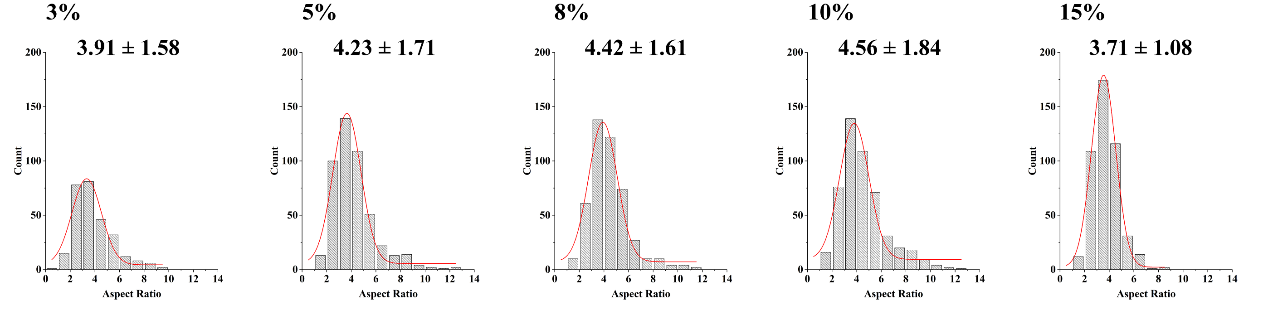


SFig. 4 Aspect ratio distribution of SrAl_12_O_19_ grain in zirconia composites with different reinforcements concentration.


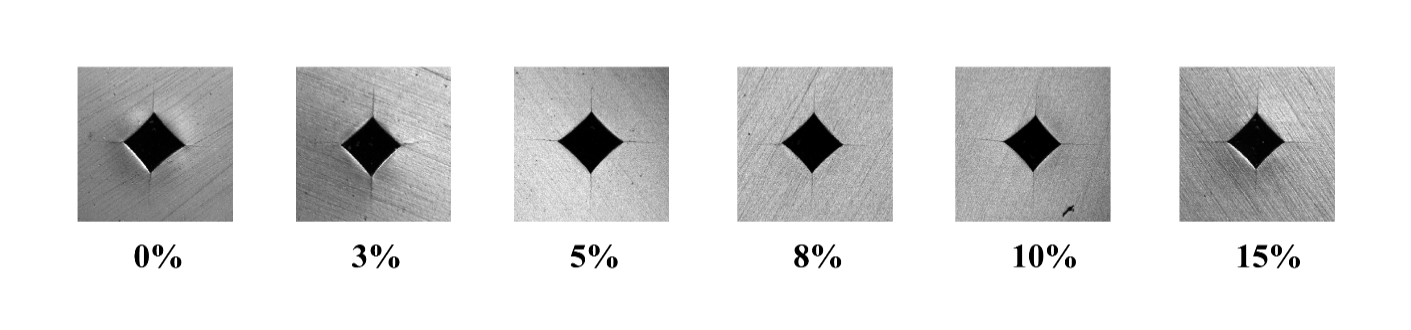


SFig. 5 The Vickers indentation in zirconia composites with different reinforcements concentration.


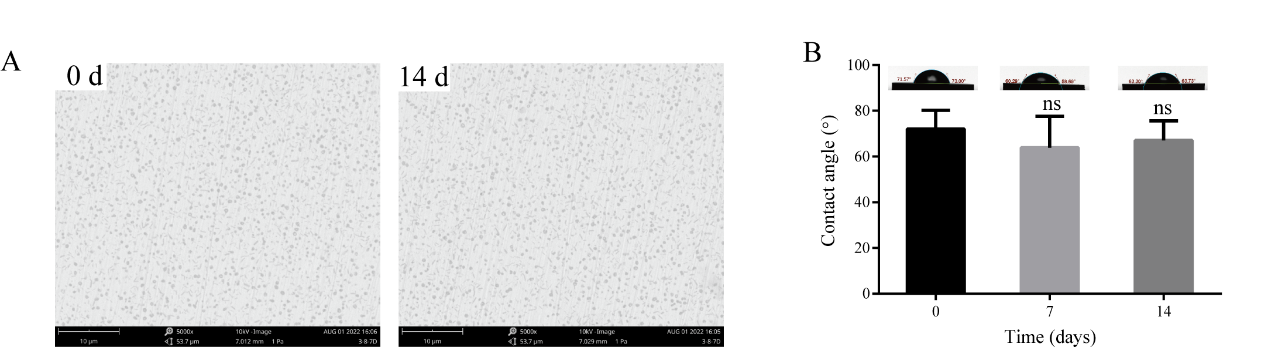


SFig. 6 SEM image and contact angles of zirconia composite after immersed for 14 days.

STable 1 Primer pairs used in the qRT‐PCR

| Gene | Primer sequence (5’-3’) |
| --- | --- |
| iNOS F | GTTCTCAGCCCAACAATACAAGA |
| iNOS R | GTGGACGGGTCGATGTCAC |
| TNF-α F | TGTCTCAGCCTCTTCTCATT |
| TNF-α R | TGATCTGAGTGTGAGGGTCT |
| CD163-F | GGTGGACACAGAATGGTTCTTC |
| CD163-R | CCAGGAGCGTTAGTGACAGC |
| CD206-F | CTCTGTTCAGCTATTGGACGC |
| CD206-R | TGGCACTCCCAAACATAATTTGA |
| FGF2-F | GCGACCCACACGTCAAACTA |
| FGF2-G | CCGTCCATCTTCCTTCATAGC |
| VEGF-F | CTGCCGTCCGATTGAGACC |
| VEGF-R | CCCCTCCTTGTACCACTGTC |
| ARG1-F | CTCCAAGCCAAAGTCCTTAGAG |
| ARG1-R | GGAGCTGTCATTAGGGACATCA |
| COL1A1-F | GAGGGCCAAGACGAAGACATC |
| COL1A1-R | CAGATCACGTCATCGCACAAC |
| COL3A1-F | GGAGCTGGCTACTTCTCGC |
| COL3A1-R | GGGAACATCCTCCTTCAACAG |
| SH3BP5-F | TATCAACCGACGGGAGACTGA |
| SH3BP5-R | TGCCAGTTCGTCTAGTTTCAC |
| GAPDH-F | AACGACCCCTTCATTGAC |
| GAPDH-R | TCCACGACATACTCAGCAC |
